# A ten-year experience of musculoskeletal tuberculosis at a tertiary care hospital in South India

**DOI:** 10.1016/j.jor.2024.05.001

**Published:** 2024-05-03

**Authors:** Druti Hazra, Rosemary Shaji V, Arushi Dhall, Arathi P. Rao, Anil K. Bhat, Kiran Chawla

**Affiliations:** a Department of Microbiology, Kasturba Medical College, Manipal, Manipal Academy of Higher Education, Karnataka, India; b Department of Radio Diagnosis, Kasturba Medical College, Manipal, Manipal Academy of Higher Education, Karnataka, India; c Department of Health Policy, Prasanna School of Public Health, Manipal, Manipal Academy of Higher Education, Karnataka, India; d Department of Hand Surgery, Kasturba Medical College, Manipal, Manipal Academy of Higher Education, Karnataka, India

**Keywords:** Musculoskeletal tuberculosis, Diagnosis, Spinal TB, Bone and joint tuberculosis, Disease management, Anti-TB treatment

## Abstract

**Background:**

The delayed identification and management of musculoskeletal tuberculosis (MSTB) poses substantial health challenges and leads to significant morbidity. This study aimed to collate ten years of hospital data and provide valuable insights into the clinical, diagnostics, and outcomes of the patients diagnosed with MSTB.

**Methods:**

A retrospective study was undertaken to review clinic records from 2013 to 2022 for all individuals diagnosed with MSTB in a tertiary care hospital in South India.

**Results:**

Over a decade, 400 cases of MSTB were diagnosed, revealing 57 % males and 43 % females with a mean age of 43.2 ± 18.9 years. Spinal TB constituted 72 % of cases, with the most common involvement of thoracic vertebrae (50.9 %). Extra-spinal MSTB accounted for 28 %, prevalent more in the pediatric age group (p < 0.05). Surgical intervention was required for 80 % of spinal TB cases and 58 % of extra-spinal MSTB cases. The average follow-up duration was two years, with 73 % completing treatment. Unfortunately, seven patients died, and three experienced relapse.

**Conclusion:**

Spinal TB is the most common type of MSTB and is predominant in young and middle-aged adults, while extra-spinal MSTB is more frequently observed in children. Where use of MRI facilitates early detection of spinal TB; histopathological and microbiological examination confirm the diagnosis. Combining anti-tubercular drugs with modern surgical approaches is essential for obtaining favorable outcomes and improving the quality of life of such patients. It is crucial to have advanced and affordable diagnostic facilities, along with increased public awareness, to reinforce tuberculosis control strategies.

## 1 Introduction

Tuberculosis (TB) is an ancient illness, detected even in several thousand-year-old Egyptian mummies with the demonstration of evidence of skeletal TB.^1^ In 2022, worldwide approximately 10.6 million TB cases were reported, and an estimated 1.3 million died.^2^ The occurrence of TB is highest in developing countries and two-thirds of the total TB cases are from eight countries, with India leading the chart (27 %).^2^ According to the India TB Report 2023, among the notified, 24 % had extra-pulmonary TB (EPTB) in 2022 and skeletal tuberculosis was the 4th most common form of EPTB.^3^ Skeletal TB is a common chronic infection of bones and marrow and approximately 50 % of all cases of skeletal tuberculosis are spinal cases.^4^^,^^5^ Musculoskeletal TB (MSTB) results from the hematogenous spread of tubercular bacilli from the primary site of infection involving the lungs or urogenital system.^6^

The manifestation of MSTB depends on the severity and location of the disease.^4^ The insidious onset of MSTB often results in delayed diagnosis, which in turn increases the risk of disease progression.^7^ A delayed diagnosis of spinal TB can lead to a higher risk of neurological deficits and spinal deformities, which may necessitate reconstructive surgery.^8^^,^^9^ Extra-spinal MSTB may present as peripheral arthritis, osteomyelitis, synovitis, tenosynovitis, or myositis.^10^ A substantial delay in diagnosing MSTB risks skeletal deformity, and disability, lowers the quality of life, as well as increases healthcare costs.^11^^,^^12^ Musculoskeletal tuberculosis remains a significant health concern in India, and understanding its clinical features, diagnosis, and outcomes is crucial for effective management. This study presents a comprehensive analysis of ten-year data on patients with musculoskeletal tuberculosis treated at a tertiary-care hospital in India. By analyzing the data, we aim to provide valuable insights into the disease's presentation, diagnostic challenges, and treatment outcomes, ultimately contributing to improved patient care and outcomes in this population.

## 2 Methods

### 2.1 Study design and settings

A retrospective study was conducted to review MSTB cases over the past ten years at a tertiary care facility in coastal Karnataka, South India. Medical records of patients diagnosed with MSTB between January 2013 and September 2022 were retrieved from the hospital's medical records system.

### 2.2 A brief description of patients’ diagnosis and management

In this study, no exclusions based on gender were made, and both adults and children were included. The signs and symptoms of the disease varied depending on the site of infection. Following the initial suspicion of TB, patients underwent radiology and routine blood investigations. Further, biopsies were collected from the infected area and assessed for histopathology and microbiology confirmation. A confirmed diagnosis was established when the samples tested positive for microbiological tests (microscopy for acid-fast bacilli (AFB)/culture/CBNAAT/PCR for MTB) or histopathology revealed granulomatous features with positive AFB. Patients were clinically diagnosed with MSTB, if samples were bacteriologically and histopathology negative, but clinic-radiological evidence was suggestive of TB, and the patient showed improvement with the administration of anti-tubercular therapy (ATT). In accordance with the TB guidelines in India, all the patients were treated with ATT and cases were notified to the Nikshay portal. Conservative drug therapy was combined with surgical management, whenever needed to improve patient outcomes. Patients were advised to have a regular follow-up to monitor the disease prognosis.

### 2.3 Ethical consideration and data collection

Foremost, we received approval from the Institutional Ethical Committee to conduct the study and access patients' medical records. To ensure accurate documentation of patients' information, a standardized proforma was created. This proforma included details such as the patient's baseline demographics, clinical characteristics, duration of symptoms, co-morbidities, history of TB, and site of infection. Additionally, all diagnostic workups including radiological, microbiological, and histopathological findings, as well as blood tests such as C-reactive protein (CRP), hemoglobin, Erythrocyte sedimentation rate (ESR), and total leucocyte counts were recorded. The radiological data of patients was retrieved and reviewed by a professional radiologist. Documentation of medical or surgical management with details of surgical procedures used, duration of hospital stays, and follow-up data was done. Patients with insufficient data were excluded from the analysis.

### 2.4 Statistical analysis

The data collected was analyzed using EZR 3.4.3, which is an open-source statistical software program. The population's characteristics were presented using descriptive statistics. Continuous variables were expressed as median with interquartile range or mean ± standard deviation (for normal distributed variables). Categorical data were presented as frequency and percentages. Additionally, the categorical and continuous data were compared using the Chi-square and T-test/Mann-Whitney tests, respectively, with statistical significance determined by p-values less than 0.05.

## 3 Results

### 3.1 Demographic and clinical characteristics

During ten years, a total of 435 cases of MSTB were recorded, out of which 35 cases were excluded from the analysis due to inadequate data. The remaining 400 patients comprised 228 (57 %) males and 172 (43 %) females, resulting in a male-to-female ratio of 1.3:1. The mean age of the patients was 43.2 ± 18.9 years, with the youngest being only 1 year old and the oldest being 87. The majority of the cases (138, 34.5 %) occurred in the age group of 40–59 years, followed by 18–39 years (133, 33.2 %), ≥60 years (90, 22.5 %), and ≤17 years (39, 9.8 %). A summary of the demographic and clinical features is presented in Table 1. In our study, there was a higher number of MSTB cases in the male group. On the other hand, extra-spinal MSTB was more common in the pediatric age group (p < 0.05).

**Table 1:** Demographic and clinical characteristics of the patients with musculoskeletal TB.

Characteristics	Total	Spinal TB	Extra-spinal MSTB	p-value
Number of cases	400	288 (72 %)	112 (28 %)	
**Gender**				
Male	228 (57 %)	153 (53 %)	75 (67 %)	p < 0.05
Female	172 (43 %)	135 (47 %)	37 (33 %)	
Mean age (years)	43.2 ± 18.9	46.3 ± 17.7	35.0 ± 19.6	p < 0.05
**Age groups (years)**				
Pediatric (≤17)	39 (9.8)	16 (5.6 %)	23 (20.5 %)	p < 0.05
Young adults (18–39)	133 (33.2)	88 (30.6 %)	45 (40.2 %)	
Middle-age adults (40–59)	138 (34.5)	110 (38.1 %)	28 (25 %)	
Senior adults (≥60)	90 (22.5)	74 (25.7 %)	16 (14.3 %)	
**Symptoms at the time of the first visit**				
Localized pain	388 (97 %)	280 (97.2 %)	108 (96.4 %)	
Swelling	70 (17.5 %)	26 (9 %)	44 (39.3 %)	p < 0.05
Low-grade fever	88 (22 %)	63 (21.9 %)	25 (22.3 %)	
Loss of weight	53 (13.3)	39 (13.5 %)	14 (12.5 %	
Neurological deficits/functional impairment	144 (36 %)	138 (48 %)	6 (5.3 %)	p < 0.05
**Co-morbidities**				
HIV	11 (2.8)	9 (3.1 %)	2 (1.8 %)	p < 0.05
Hepatitis B virus	8 (2 %)	6 (2 %)	2 (1.7 %)	
Concomitant pulmonary TB	89 (22.2 %)	68 (23.6 %)	21 (18.7 %)	
Diabetes	62 (15.5 %)	51 (17.7 %)	11 (9.8 %)	
Hypertension	54 (13.5 %)	44 (15.3 %)	10 (8.9 %)	
Chronic kidney disease	11 (2.8)	7 (2.4 %)	4 (3.6 %)	
Duration of symptoms in days				
Median (IQR)	90 (30, 180)	90 (30, 180)	60 (30, 180)	

Among MSTB patients, localized pain (388, 97 %), low-grade fever (88, 22 %), and weight loss (53, 13.3 %) were the most common clinical symptoms. Patients with spinal TB had a higher incidence of neurological deficits (138, 48 %) compared to extra-spinal MSTB patients (6, 5.3 %) (p < 0.05). In contrast, persistent swelling (44, 39.3 %) was more common in extra-spinal MSTB cases compared to spinal TB (26, 9 %) (p < 0.05). The median duration from the onset of symptoms to visit a tertiary care hospital was 90 (30,180) days.

Among the patients evaluated, the most frequent comorbidity observed was diabetes, affecting 15.5 % (62) of the individuals, followed by hypertension (54, 13.5 %), and chronic kidney disease (11, 2.8 %). A total of 11 patients (2.8 %) tested positive for Human Immunodeficiency Virus (HIV), while 8 patients (2 %) were infected with Hepatitis B virus. Moreover, pulmonary TB was diagnosed in 89 cases (22.2 %), while pleural TB was found in 10 (2.5 %) patients. Disseminated tuberculosis was observed in six patients (1.5 %), five patients (1.3 %) exhibited lymph node tuberculosis, one had endometrial tuberculosis, and another had peritoneal tuberculosis.

### 3.2 Radiology findings

**Fig. 1:**
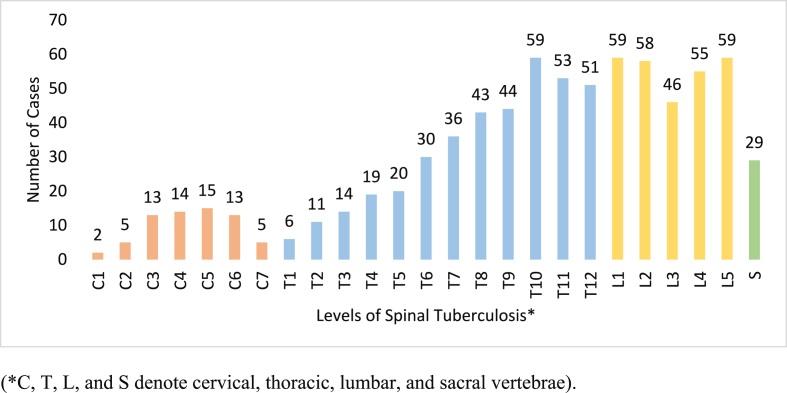
The distribution of vertebral involvement at each spinal level in cases of spinal tuberculosis.

After undergoing clinical evaluation, all suspected cases of MSTB received an X-ray examination. Further evaluation was conducted using MRI and/or CT scans in 272 (68 %) patients to obtain more details on the spread of the infection through the skeleton, organs, and soft tissues. Among the 400 MSTB cases, 288 (72 %) were diagnosed with spinal TB. The thoracic vertebrae (386, 50.9 %) were the most frequently affected, followed by the lumbar (277, 36.5 %), cervical (67, 8.8 %), and sacral (29, 3.8 %) region [Fig. 1]. Additionally, vertebral skipped lesions were observed in 27 (9.4 %) cases [Table 2]. According to the imaging study, 112 (38.9 %) patients had paraspinal abscesses and 36 (12.5 %) patients had kyphotic deformity.

**Table 2:** Anatomical sites of musculoskeletal tuberculosis infection.

Anatomical sites	Frequency (n)	Percentage (%)
**Spinal TB location**		
Cervical	67	8.8
Thoracic	386	50.9
Lumbar	277	36.5
Sacral	29	3.8
**Spinal skipped lesion**		
C, L	2	0.7
C, T	6	2.0
C, T, L	1	0.3
T, L	12	4.2
T, L, S	3	1.0
T, T	3	1.0
**Number of vertebral bodies involved**		
1	12	4.2
2	182	63.2
≥3	94	32.6
**Extra-spinal TB location**		
Sacro iliac joint	6	1.5
Hip	18	4.5
Knee	28	7
Ankle	12	3
Sternoclavicular joint	3	0.7
Elbow	5	1.3
Shoulder	3	0.7
Leg (Femur & Tibia)	13	3.3
Arm (Humerus, Radius, Ulna)	5	1.3
Foot	9	2.2
Hand	4	1
Sternum	4	1
Clavicle	2	0.5

Out of all included cases, 112 (28 %) cases had extra-spinal MSTB. Among these, the knee with 28 cases (7 %), experienced the highest incidence, followed by the hip (18, 4.5 %), leg (13, 3.3 %), and ankle (12, 3 %). According to imaging reports, 35 (31.2 %) of these cases had osteomyelitis, followed by arthritis (32, 28.5 %), synovitis (31, 27.7 %), sacroiliitis (6, 5.4 %), tenosynovitis (6, 5.4 %) and dactylitis (2, 1.7 %). Abscess formation was noted in 25 % of extra-spinal MSTB cases.

### 3.3 Laboratory diagnosis

In histopathological analysis, TB-suggestive features like epithelial cell granulomas, Langerhans giant cells, and caseating necrosis were present in 79 % (276/349) of the MSTB cases. Out of the samples tested, 82.8 % (271/327) were positive for Xpert MTB/RIF or polymerase chain reaction (PCR) for MTB. MGIT cultures were positive for 60.1 % (80/133) of the samples, and AFB was positive for 35 % (100/268) under fluorescence microscopy. A mixed infection of *Mycobacterium tuberculosis* and *M. abscessus* was noted in a 64-year-old male patient.

The patients' routine laboratory test results showed that the median erythrocyte sedimentation rate (ESR) was 49 (25, 72) mm/hour. Additionally, their median hemoglobin levels were within the normal range at 12 (10, 13) gm/dl, and their mean white blood cell (WBC) count was 9.3 ± 3.7 × 10^3^/μL. The median C-reactive protein (CRP) value was 24 (7, 45) mg/L.

### 3.4 Drug-resistant TB

Ten cases of drug-resistant tuberculosis (DR-TB) were identified through the Line-probe assay and liquid culture drug-susceptibility test (DST) among the reported instances. Two of the patients were mono-resistant to rifampicin (RIF) and one to pyrazinamide (PZA). Five cases were multi-drug resistant TB (MDR-TB), resistant to rifampicin and isoniazid (INH). Three cases of pre-extensively-drug resistant TB (Pre XDR-TB) were also detected.

### 3.5 Treatment and outcomes

Among the 288 spinal TB patients, 56 (20 %) were chosen for conservative treatment, and surgical management was required for 232 (80 %) cases based on the severity of the disease. 54 patients (18.7 %) underwent non-instrumented surgeries such as abscess evacuation, spinal compressive element removal, and wound debridement followed by prolonged immobilization. Instrumented surgeries were performed on 163 cases (56.6 %), with 49 patients (17 %) undergoing vertebral fusions. Out of 112 cases of extra-spinal MSTB, 65 (58 %) underwent surgery. The types of surgeries performed included synovectomy (10.7 %), arthrotomy (7.1 %), arthroplasty (3.6 %), and bone grafting (1.8 %). Other surgical interventions like debridement, curettage, and abscess drainage were performed on 26.8 % of the patients.

On average, patients were followed up for two years. Of the total 400 patients, 292 (73 %) completed the treatment successfully. Seventy-three (18.2 %) patients were lost to follow-up, while twenty-five (6.2 %) patients are still receiving anti-tubercular treatment. Unfortunately, seven patients died, and three patients experienced a relapse, resulting in unfavorable treatment outcomes.

### 3.6 Trend of musculoskeletal TB from 2013 to 2022

**Fig. 2:**
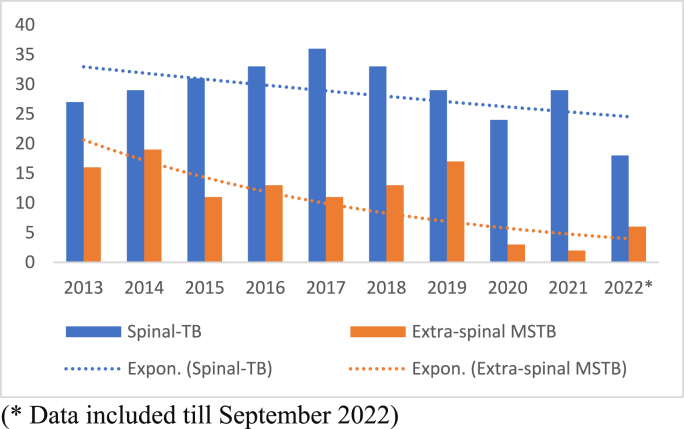
Trend of Spinal and extra-spinal musculoskeletal TB cases from 2013 to 2022.

In this current study, we observed a gradual declining trend in diagnosed cases of MSTB, particularly in extra-spinal instances (Fig. 2). The number of cases was slightly lower in 2020, likely due to the impact of the COVID-19 pandemic, as health resources for TB diagnostic services were redirected to address the COVID-19 crisis.

## 4 Discussion

Despite significant efforts to contain the spread of tuberculosis over the past few decades, it remains a major public health concern around the world. Although the incidence of MSTB is relatively low, it can cause significant functional impairment, making it a major healthcare challenge even in modern times. In this study, the majority of MSTB patients were young and middle-aged, with 67.7 % of cases between 18 and 59 years old. Previous studies conducted in endemic TB countries, such as India, China, and South Africa, also reported a higher incidence of MSTB in individuals aged between their third and fifth decade of life.^13^, ^14^, ^15^ In developed nations, MSTB is more prevalent among the native population over 60 years old.^16^, ^17^, ^18^ Moreover, in this study, children under the age of 17 years had a relatively higher incidence of extra-spinal MSTB than spinal tuberculosis. The bones of children under 18 years of age have increased vascularity, making them more vulnerable to hematogenous infections that can spread to the distal bone shaft, end, and nearby joint space from the initial site of infection.^19^ In our study, 57 % of diagnosed MSTB cases were male, consistent with the male predominance observed in previous research.^20^^,^^21^ Nonetheless, Kori et al. underscored a higher prevalence of females in urban slums and rural areas of Central India, emphasizing the urgent need for targeted interventions to combat tuberculosis within vulnerable populations.^13^ Musculoskeletal tuberculosis typically emerges as a delayed consequence of the spread through the bloodstream or lymphatic system and often manifests several years after the initial infection. In our clinical setting, the spine exhibited the highest incidence of MSTB involvement, representing 72 % of cases. Consistent with prior research,^18^^,^^22^ the thoracic vertebra was most frequently affected (50.9 %), followed by the lumbar vertebra (36.5 %). However, few other studies have also reported the lumbar segment as a commonly affected site.^23^^,^^25^ Besides the spine, tuberculosis affected peripheral joints or bones in 28 % of patients, with the knee (7 %), hip (4.5 %), leg (3.3 %), and ankle (3 %) being commonly involved. Similarly, Ozdemir et al. noted 25 % of extraspinal MSTB cases, with a higher incidence observed in weight-bearing bones and joints, possibly attributable to increased vascularization in these areas.^14^^,^^26^ Furthermore, in this study, 112 patients (28 %) exhibited extra-skeletal TB infection, with 89 cases (22.2 %) demonstrating MSTB concurrent with pulmonary TB, followed by pleural TB (10, 2.5 %). MSTB is frequently secondary to pulmonary TB and may coincide with other forms of extra-pulmonary TB. Hence, early diagnosis and prompt treatment of pulmonary TB are crucial to curb further infection spread and prevent secondary MSTB deformity.^21^

The differential diagnosis of MSTB is guided by clinical and radiological examinations, including X-rays, CT scans, and MRI imaging. Conventional X‐ray radiograph is a relatively affordable and easily accessible technique to assess the extent of damage in the affected area. However, X-ray findings in MSTB are nonspecific and could be normal at the earliest stage of the disease, thus a further diagnostic evaluation is required.^27^ Advanced imaging techniques like MRI and CT are still the most useful to provide diagnostic features of MSTB. In our study, an X-ray radiograph was available for all patients, and for the majority of the patients (68 %), MRI and/or CT scans were available. MRI is a highly sensitive tool particularly useful for demonstrating vertebral changes, intraosseous and paraspinal soft tissue abscesses, cord compression, cord changes, and skipped lesions which are suggestive of infective TB etiology.^28^^,^^29^ CT remains the preferred imaging modality for the assessment of osteomyelitis showing cortical bone destruction and sequestration formation and for guided aspiration cytology in patients in whom the diagnosis is uncertain.^29^

Post-clinico-radiological examination, histopathological and microbiological evaluations should be conducted for the definitive diagnosis of MSTB. Histopathological diagnosis of TB from tissue samples often requires the presence of classical features of caseous necrosis, granuloma, and the presence of acid-fast bacilli. In this present study, histopathological suggestive features of TB were noted in 79 % of cases, whereas previous studies reported correlations ranging from 46.5% to 97 %.^22^^,^^30^^,^^31^ A false-negative histopathological finding can occur due to sampling errors or non-uniform distributions of microorganisms. Culturing TB bacilli is regarded as the gold standard for definitively diagnosing TB. Previous studies have reported MTB culture positivity rates ranging from 19.2 % to 87 %,^18^^,^^30^^,^^32^ while our study found a 60 % culture-positive rate. The paucibacillary nature of the disease and technical errors frequently diminish cultural yield. Additionally, microbiological specimens should be sent formalin-free to avoid false negatives, as formalin can kill any present organisms.^11^ Utilizing advanced diagnostic tools such as GeneXpert MTB/RIF Ultra has significantly enhanced the sensitivity of detecting Mycobacterium tuberculosis (MTB) and rifampicin (RIF) resistance. Our study demonstrated a positivity rate of 82.8 % for MTB using GeneXpert or PCR techniques. A meta-analysis conducted by Shen et al. reported a remarkable 96 % sensitivity and 85 % specificity of Xpert MTB/RIF compared to culture, establishing it as a highly reliable tool for diagnosing MSTB.^33^

The early detection and management of MSTB is crucial to prevent skeletal deformity, neural dysfunction, and poor prognosis of affected patients. In our study, we found that 80 % of patients with spinal tuberculosis underwent corrective surgeries, consistent with previous research conducted in Asia.^24^^,^^34^ Furthermore, our analysis revealed that 58 % of cases presenting with extra-spinal skeletal TB underwent surgical intervention, a notably higher proportion compared to the 12.7 % reported by Barik et al. from India.^31^ This is likely because our hospital is a tertiary care center, where patients with advanced disease are often referred for comprehensive disease management. The role of surgery in peripheral bone and joint tuberculosis is limited and has not been studied as extensively as in spinal tuberculosis. A combination of surgery and chemotherapy can lead to significant improvement in both short- and long-term outcomes. Therefore, depending on the disease severity in different patients, the selection of appropriate surgical procedures can achieve individualized treatment. A future prospective randomized controlled trial with a larger sample is necessary to provide enhanced information on the management of musculoskeletal TB.

## 5 Conclusion

Musculoskeletal TB exhibits a notably nonspecific clinical presentation. In regions with a high prevalence of TB, clinicians must maintain a high index of suspicion and awareness for MSTB diagnosis. Spinal TB, predominantly affecting young and middle-aged adults, represents the most common form of MSTB, while extra-spinal MSTB is more prevalent in children. Utilizing MRI can facilitate earlier diagnosis, and all clinically suspected patients should undergo confirmation by histopathological and microbiological diagnosis. Modern diagnostic techniques like Xpert MTB/RIF Ultra offer a rapid diagnosis for MSTB. Combining anti-tubercular drugs with contemporary surgical management is essential for favorable outcomes, improving patients' quality of life. The expansion of advanced diagnostic facilities at an affordable cost and enhanced public awareness of the disease are imperative for reinforcing tuberculosis control strategies.

## Funding

No funding was received for conducting this study.

## Declaration of patient's consent

Not Applicable.

## Ethical approval

The study was approved by the Kasturba Medical College and Kasturba Hospital Institutional Ethics Committee [IEC: 374/2022 and IEC: 230/2018].

## Patient's consent

Consented to participate does not apply to this retrospective study.

## CRediT authorship contribution statement

**Druti Hazra:** Methodology, Software, Data curation, Visualization, Writing – original draft. **Rosemary Shaji V:** Methodology, Writing – review & editing. **Arushi Dhall:** Methodology, Writing – review & editing. **Arathi P. Rao:** Writing – review & editing. **Anil K. Bhat:** Conceptualization, Supervision, Writing – review & editing. **Kiran Chawla:** Conceptualization, Methodology, Supervision, Writing – review & editing.

## Declaration of competing interest

The authors declare that they have no conflict of interest.
